# Recent advances in understanding genetic variants associated with growth, carcass and meat productivity traits in sheep (*Ovis aries*): an update

**DOI:** 10.5194/aab-62-579-2019

**Published:** 2019-10-23

**Authors:** Alexander S. Zlobin, Natalia A. Volkova, Pavel M. Borodin, Tatiana I. Aksenovich, Yakov A. Tsepilov

**Affiliations:** 1 Institute of Cytology and Genetics, Siberian Branch of the Russian Academy of Sciences, Novosibirsk, Russia; 2 L. K. Ernst Federal Science Center for Animal Husbandry, Dubrovitsy, Moscow Region, Russia; 3 Novosibirsk State University, Novosibirsk, Russia

## Abstract

Identification of quantitative trait loci (QTLs) and candidate genes that affect growth intensity is a prerequisite for the marker-assisted selection of economically important traits. The number of QTL studies on sheep is relatively small in comparison to those on cattle and pigs. The current QTL sheep database – Sheep QTLdb – contains information on 1658 QTLs for 225 different traits. A few genes and markers associated with growth, carcass and meat productivity traits have been reported. The information about QTLs from the Sheep QTLdb cannot be directly used in marker-assisted selection due to the lack of essential information such as effective and reference alleles, the effect direction etc., and it requires manual curation and validation. In this study we performed a comprehensive search for QTLs focusing on single nucleotide polymorphisms (SNPs) associated with growth and meat traits in sheep. The database contains information about 156 SNP–trait associations (123 unique SNPs) and a list of 165 associated genes. The updated information is freely available at https://github.com/Defrag1236/Ovines_2018 (last access: 18 September 2019). This information can be useful for further association studies and preliminary estimation of genetic variability for economically important traits in different breeds.

## 1 Introduction

Identification of quantitative trait loci (QTLs) and candidate genes that affect growth traits is a prerequisite for the marker-assisted selection of economically important traits. Since the 1990s when QTL mapping on farm animals was initiated, thousands of QTLs have been identified for a large number of traits (Hu et al., 2013). The number of QTL studies on sheep is relatively small in comparison to those on cattle and pigs. The current QTL sheep database – Sheep QTLdb – contains information on 1658 QTLs for 225 different traits (Hu et al., 2016). Recently, Xu and Li (2017) reviewed information about markers associated with economically important traits in sheep revealed by high-throughput screening technologies. A few genes and markers associated with growth, carcass and meat productivity traits were reported.

Unfortunately the information about QTLs from the Sheep QTLdb cannot be directly used in marker-assisted selection due to the lack of essential information (effective and reference alleles, the effect direction etc.), and it requires manual curation and validation. The estimation of variability of associated single nucleotide polymorphisms (SNPs) in local sheep breeds as well as in closely related wild ovine species can provide valuable information for selective improvement of the existing breeds and generation of resource populations.

The aim of this study was to generate the most recent and comprehensive list of genes and SNP markers associated with growth, carcass and meat productivity traits in sheep.

## 2 Material and methods

### 2.1 Search and selection of scientific papers

For searching and filtering we used PubMed (https://www.ncbi.nlm.nih.gov/pubmed, last access: 18 October 2019) and Google Scholar (http://scholar.google.com, last access: 18 October 2019). We restricted our search to papers published in English in peer-reviewed journals since 2013. We chose 2013 as the year when the first biggest genome-wide association study (GWAS) (n>200) on growth and meat production traits in sheep was published (Zhang et al., 2013). Keyword combinations included words related to organism (sheep, ovine, *Ovis aries*), to trait of interest (birth weight, weaning weight, 6-month weight etc.), to type of study (GWAS, gene candidate, association study). For example, one of the keyword combinations was “sheep growth production traits GWAS”. All used keyword combinations are shown in table ST1 in the Supplement. For each keyword combination we manually screened and selected the top 20 most relevant papers for each database for further consideration.

Next, we selected the earliest (starting from 2013) and the most relevant papers describing GWAS of growth and meat production traits in sheep (Zhang et al., 2013; Wang et al., 2015; Kominakis et al., 2017). We assume that GWASs that contain information about several relevant associated QTLs and candidate genes potentially have larger citation trees than gene candidate papers. We traced the citation trees of these papers in Google Scholar, selected the most relevant papers and checked them for overlap with the papers found at the first stage (see Supplement Table ST2).

All papers selected previously were manually organized by two researchers and filtered by relevance. At this stage we extracted information from papers such as year of publication, journal name, studied trait, sample size, breed, type of study etc. All this information is summarized in Supplement Table ST3. We graded all papers by relevance. We used following grades: 1 (the most relevant), 2 (still relevant, but does not contain exact information about the effect sizes, effective and reference alleles, or p values) and 3 (not relevant or search mistake). Papers with a score of 1 or 2 were considered in the final list of publications to be analysed.

### 2.2 Extraction of genes and QTLs

Two researchers independently extracted the following information: (1) the list of associated SNPs with all available information, including SNP name, chromosome and position, effective (allele for which the effect was estimated) and reference alleles, effect size and standard error, frequency, nearest or prioritized gene, and – if a gene candidate – forward and reverse primer sequences; (2) the list of prioritized genes. If publication consisted of genome-wide significant and chromosome-wide significant or suggestively significant QTLs, we selected the genome-wide significant only. For each gene from the gene list we calculated the score as the number of publications (without reviews) reporting this gene.

## 3 Results

### 3.1 Search and selection of scientific papers

Using 15 different keywords combinations (see Table ST1) we found 153 papers (including duplicates). We discovered that the words related to organism and trait of interest in the keyword combinations used for the search are more important than the word related to the type of the study. Next, we investigated the citation tree of the paper of Zhang et al. (2013) in Google Scholar and related papers on PubMed. We found 16 papers, 9 of which were absent in ST1 (see Table ST2). Next we selected two papers (Wang et al., 2015; Kominakis et al., 2017) and repeated citation tree analysis that led to one additional paper (Table ST2). In this stage after removing duplicates we had 46 papers to examine in detail.

Next, all the found papers were manually organized by two researchers and filtered by the relevance. We selected the most relevant papers (see Fig. S3) that led to the final list of 18 publications. Nine of them described GWAS analysis (Riggio et al., 2013; Zhang et al., 2013; Al-Mamun et al., 2015; Bolormaa et al., 2016; Matika et al., 2016; Kominakis et al., 2017; Garza Hernandez et al., 2018; Pasandideh et al., 2018; Ghasemi et al., 2019), seven described gene candidate analysis (Jalil-Sarghale et al., 2014; Cinar et al., 2016; Ma et al., 2016; Trukhachev et al., 2016; Zhang et al., 2016; Aali et al., 2017; Armstrong et al., 2018) and two were reviews (Sahu et al., 2017; Xu and Li, 2017) (Table ST3).

### 3.2 Extraction of genes and QTLs

From the finally selected 18 papers we extracted information about associated genes and QTLs (SNPs). A total 198 genes (172 unique genes) were associated with different growth, carcass and meat production traits (Table ST4). A common tendency to report only the nearest gene to the SNP found can lead to a bias in interpretation: the functional gene (which is casually associated with the trait of interest) is not necessarily the nearest to the SNP (Pers et al., 2015).

We extracted information about associated SNPs with all available information (effect sizes, effective and reference alleles, etc.). In total we found information about 163 SNP–trait associations (130 unique SNPs). Reported SNPs were presented in different formats, such as array format (e.g. OAR21_7449077.1), rs name, custom name for gene candidate studies (e.g. SNP23) or no name (only chromosome and position). Unfortunately, only 5 out of 17 papers contained information about the effective alleles (4 gene candidate studies and 1 GWAS; Jalil-Sarghale et al., 2014; Ma et al., 2016; Trukhachev et al., 2016; Zhang et al., 2016; Armstrong et al., 2018) Only three papers reported the effect sizes for associated SNPs (Al-Mamun et al., 2015; Armstrong et al., 2018; Garza Hernandez et al., 2018). A total of 89 out of 130 associated SNPs were present in the Ovine Infinium^®^ HD SNP Bead-Chip array (606 K).

## 4 Discussion

We generated a new database for growth and daily gain traits published since 2013. It consists of 17 papers, twice more than Sheep QTLdb (Hu et al., 2016). Of course, there are still a rather small number of publications in comparison to the abundant human genetic data. Using the same filters and keywords “human height traits GWAS”, one can find 5 times more articles (98).

After rigorous manual extraction of all information about associated regions, we came up with the list of 172 unique genes associated with growth, carcass and meat productivity traits. We estimated the gene scores (number of publication where this gene was reported) using only GWAS papers, because they provide a hypothesis-free search, and we excluded gene candidate papers because a substantial part of these studies is based on previous GWASs. In GWASs (eight publications in total) only three genes had a score of > 1: *ACACA* (chromosome 11), *NCAPG* and *LCORL* (chromosome 6). We also performed gene set enrichment and tissue-specific analysis (see .docx file from Supplement) but did not observe any specific enrichment or clusters.

Gene *ACACA* encodes protein that catalyses the rate-limiting reaction in the biogenesis of long-chain fatty acids. This candidate gene was reported to be associated with muscle depth, fatty acid formation and several multivariate phenotypes related to carcass (Bolormaa et al., 2016; Garza Hernandez et al., 2018).

Gene *NCAPG* encodes protein CAP-G, which is a subunit of condensin I, a large protein complex involved in chromosome condensation. It was associated with body weight, post-weaning gain, bone-related traits and multivariate phenotype (Al-Mamun et al., 2015; Bolormaa et al., 2016).

Gene *LCORL* encodes a transcription factor that appears to function in spermatogenesis. It was associated with body weight, post-weaning gain, bone-related traits and several carcass multivariate phenotypes (Al-Mamun et al., 2015; Bolormaa et al., 2016).

Such a low number of genes overlapping between different studies can be attributed to the tendency towards reporting only the nearest-to-SNP gene. Gene prioritization refers to a family of computational techniques for inferring which gene from a studied region (if there are more than one) is really associated with trait of interest. Only two papers have performed the specific gene prioritization techniques (Bolormaa et al., 2016; Kominakis et al., 2017). Larger sample sizes and more advanced approaches for gene prioritization are necessary to improve the concordance between the reported genes.

We have collected the information about 163 SNP–trait pairs (130 unique SNPs). Only three papers contain information about the size of the allele effect. Five papers contained information about reference/effective allele. This information is essential for practical applications to marker-assisted selection. It could help to estimate the prevalence of “favourable” alleles that improve economically important traits in given populations. This indicates a necessity for sheep researcher community to follow the standards of reporting the results of association accepted in cattle or human genetics (Winkler et al., 2014; Reed et al., 2015). For each association the effective/reference allele, chromosome and position, effect size and standard error of effect size, effective allele frequency should be reported. This will improve the quality of the studies and make them more useful for practical application.

## 5 Conclusions

In this study we performed a comprehensive search of published papers that contain information about markers associated with growth and meat traits in sheep. The database contains information about 163 SNP–trait associations (130 unique SNPs) and a list of 172 associated genes. The updated information is freely available at https://github.com/Defrag1236/Ovines_2018. This information can be useful for further association studies and preliminary estimation of genetic variability in different breeds.

## Supplement

10.5194/aab-62-579-2019-supplement The supplement related to this article is available online at: https://doi.org/10.5194/aab-62-579-2019-supplement.

## Data Availability

The recent updated information is freely available at (Zlobin et al., 2019).
