# Supplementary material for: Recent advances in understanding genetic variants associated with growth, carcass and meat productivity traits in sheep (Ovis aries): an update
Source: Arch Anim Breed. 2019 Oct 23;62(2):579–83. doi: 10.5194/aab-62-579-2019 (PMC6904904; doi:10.5194/aab-62-579-2019)
Supplement: The supplement related to this article is available online at: https://doi.org/10.5194/aab-62-579-2019-supplement. [file aab-62-579-supplement.zip › aab-62-579-2019-supplement-title-page.pdf]

Supplement of Arch. Anim. Breed., 62, 579–583, 2019
https://doi.org/10.5194/aab-62-579-2019-supplement
© Author(s) 2019. This work is distributed under
the Creative Commons Attribution 4.0 License.

Open Access
Archives Animal Breeding

Supplement of

Recent advances in understanding genetic variants associated with growth, carcass and meat productivity traits in sheep (*Ovis aries*): an update

Alexander S. Zlobin et al.

Correspondence to: Yakov A. Tsepilov (tsepilov@bionet.nsc.ru)

- aab-62-579-2019-supplement-title-page.pdf
- 20190225_gene_set_enrichment.docx
- 20190610_Supplementary_tables for_Ovines_2018_paper.xlsx

The copyright of individual parts of the supplement might differ from the CC BY 4.0 License.
