# Supplementary material for: Recent advances in understanding genetic variants associated with growth, carcass and meat productivity traits in sheep (Ovis aries): an update
Source: Arch Anim Breed. 2019 Oct 23;62(2):579–83. doi: 10.5194/aab-62-579-2019 (PMC6904904; doi:10.5194/aab-62-579-2019)
Supplement: The supplement related to this article is available online at: https://doi.org/10.5194/aab-62-579-2019-supplement. [file aab-62-579-supplement.zip › 20190225_gene_set_enrichment.docx]

**Supplementary Note**

We perform gene set enrichment analysis for set of 165 genes (ST4 table). We used GSEA Panther (<http://pantherdb.org/>) tool.

In GSEA Panther we selected *Bos taurus*, because *Ovis aries* doesn’t presented in database. The results are presented in Figure 1 and Figure 2. Figure 1 shows distribution of genes between different categories of their molecular function (Figure 1).


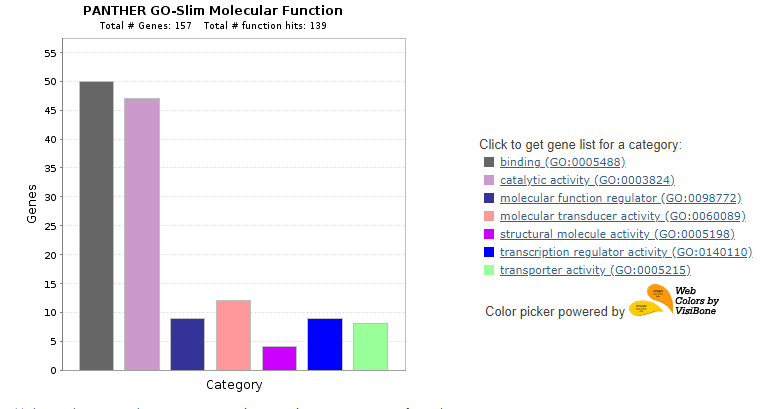
**Figure 1.** Distribution of genes between different categories of their molecular function (GSEA Panther database).

We observed the biggest part of genes (more than 45) involved in groups of binding and catalytic activity. Figure 2 shows distribution of genes between different categories of their biological process. We observed two highest signals: biological regulation and metabolic process. We did not observed any specific enrichment. The highest enrichment represent very general categories.


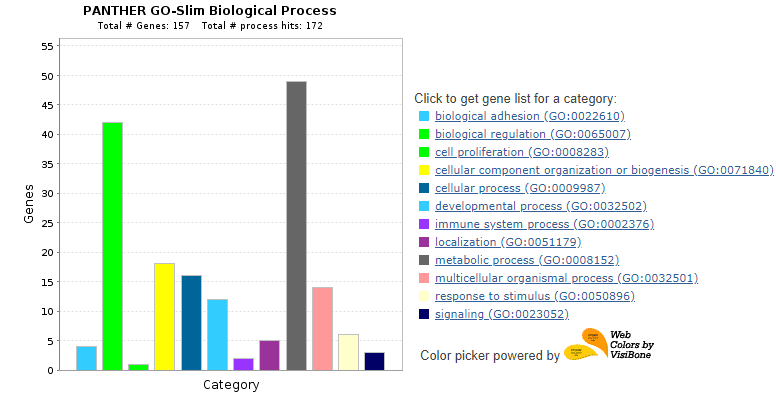
**Figure 2.** Distribution of genes between different categories of their biological process (GSEA Panther).

Moreover we performed tissue specific expression analysis using Expression Atlas (<https://www.ebi.ac.uk/gxa/home>). This tool contains information about 6 sheep studies. We used 165 genes (ST4 table) for analysis. Results are presented in Figure 3. We also did not observe any specific clusters of expression.


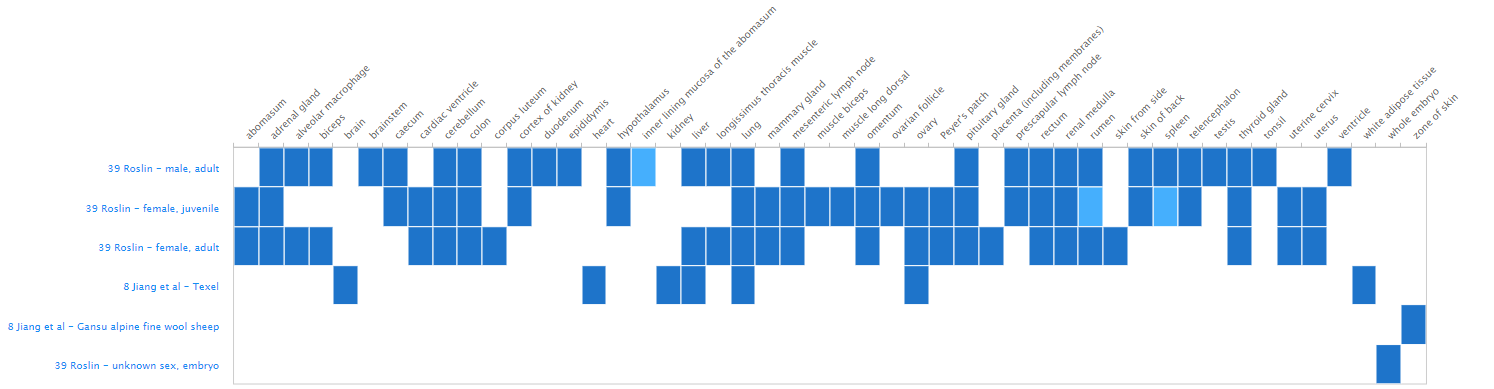


**Figure 3.** Results of tissue specific expression analysis (Atlas Expression).
